# Social Cognition in a Research Domain Criteria Perspective: A Bridge Between Schizophrenia and Autism Spectra Disorders

**DOI:** 10.3389/fpsyt.2020.00806

**Published:** 2020-08-28

**Authors:** Stefano Barlati, Alessandra Minelli, Anna Ceraso, Gabriele Nibbio, Rosana Carvalho Silva, Giacomo Deste, Cesare Turrina, Antonio Vita

**Affiliations:** ^1^Department of Clinical and Experimental Sciences, University of Brescia, and Department of Mental Health and Addiction Services, ASST Spedali Civili of Brescia, Brescia, Italy; ^2^Genetics Unit, IRCCS Istituto Centro San Giovanni di Dio Fatebenefratelli, Brescia, Italy; ^3^Department of Molecular and Translational Medicine, University of Brescia, Brescia, Italy; ^4^Department of Clinical and Experimental Sciences, University of Brescia, Brescia, Italy; ^5^Department of Mental Health and Addiction Services, ASST Spedali Civili of Brescia, Brescia, Italy

**Keywords:** schizophrenia spectrum disorder, autism spectrum disorder, neurodevelopmental disorders, social cognition, research domain criteria (RDoC) neuroimaging, genetic

## Abstract

Schizophrenia and autism spectra disorders are currently conceptualized as distinct clinical categories. However, the relationship between these two nosological entities has been revisited in recent years due to the evidence that they share some important clinical and neurobiological features, putting into question the nature and the extent of their commonalities and differences. In this respect, some core symptoms that are present in both disorders, such as social cognitive deficits, could be a primary target of investigation. This review briefly summarizes the commonalities and overlapping features between schizophrenia and autism spectra disorders in social cognitive functions, considering this construct in a Research Domain Criteria perspective. The clinical manifestation of deficits in social cognition are similar in schizophrenia spectrum disorders and autism spectrum disorders, and brain areas that appear to be altered in relation to these impairments are largely shared; however, the results of various studies suggest that, in some cases, the qualitative nature of these alterations may be different in the two spectra. Moreover, relevant differences could be present at the level of brain networks and connections. More research is required in this field, regarding molecular and genetic aspects of both spectra, to better define the neurobiological mechanisms involved in social cognition deficits, with the objective of developing specific and targeted treatments.

## Introduction

Social cognition (SC) can be broadly defined as a domain encompassing all the cognitive processes related to interpersonal contacts and to the perception of oneself and others in the social environment (1, 2). It includes a wide range of abilities, from basic ones such as recognition and processing of emotions in facial expressions and tones of voice, to more complex skills involving the attribution of mental states or the perception and understanding of social cues and contexts. These processes regulate and determine social behaviors and are closely linked to interpersonal relationships and social functioning (3).

SC currently represents a prominent field of study in Schizophrenia Spectrum Disorders (SSD), as deficits in socio-cognitive performance are related to poorer functional capacity and community-living skills, worse real-world functioning and lower quality of life (4–9). The socio-cognitive processes that appear to be most commonly impaired in patients diagnosed with SSD are emotional processing, social perception, attributional style, and Theory of Mind (ToM) (7, 10–12); these deficits may predate the clinical onset of the disorder and appear to be present since the early phases of illness, remaining substantially stable afterward (13–16).

Autism Spectrum Disorders (ASD) also represent a category of conditions characterized by significant impairments in interpersonal understanding and behaviors, with atypical social interactions and communication (17, 18). Social isolation and community-living impairment resulting from these socio-cognitive deficits are common features in individuals with ASD (19–21), often leading to lower quality of life (22–24). These SC deficits appear to have an impact on functional and social skills in subjects with ASD also in the presence of a normal Intelligence Quotient (IQ) (25).

The aim of the present narrative and critical review is to provide an overview of clinical, neuroanatomical-neurofunctional and molecular features involved in socio-cognitive deficits across the SSD and ASD spectra, highlighting how implementing knowledge in these fields in a Research Domain Criteria (RDoC) perspective could represent a valid step in improving the management and the treatment of these disorders. In fact, the issue of overlaps between SSD and ASD has not yet been explored in a RDoC perspective: filling this current gap could improve the understanding of interactions between neurobiological and clinical observations and further the integration of recent scientific knowledge into daily clinical practice.

### Schizophrenia and Autism Spectra Disorders: Areas of Clinical Overlap

SSD and ASD are currently conceptualized as separate nosological entities, emerging at different developmental periods and characterized by specific and distinctive features (26). However, this dichotomic separation has been recently called into question, and the areas of overlap between the two spectra have become the focus of a growing body of literature (27–32).

ASD symptoms are more frequent in subjects diagnosed with SSD than in healthy controls (33, 34), and appear to play a relevant role in the clinical situation of patients with SSD, as more severe ASD symptoms represent an individual predictor of worse SC performance (35, 36) and poorer real-world social functioning (37), and are correlated with greater impairments in the ability to judge the quality of everyday functioning (38). Individuals diagnosed with SSD and showing prominent ASD features could represent a particular sub-population with specific clinical characteristics, including lower IQ and poorer cognitive performance (39, 40) and worse response to antipsychotic treatment (41).

On the other hand, psychotic features are frequent in subjects diagnosed with ASD (42, 43). Childhood ASD features and ASD diagnosis are associated with psychotic experiences (44) and with substantially increased risk of SSD (45, 46). Moreover, individuals with ASD and prominent psychotic features appear to represent a peculiar sub-population, characterized by fewer stereotyped interests and behaviors and lower IQ (47).

A recent meta-analysis comparing non-social cognitive profiles of subjects diagnosed with SSD and ASD reported important differences between the disorders regarding deficits in visuospatial perception and reasoning and problem solving domains; however, differences in working memory and language performance were small, and a substantial overlap was observed in processing speed and verbal comprehension domains (48).

### Social Cognition: A Bridge Between Schizophrenia and Autism Spectra Disorders

Deficits in SC in particular represent a key feature of both spectra (13, 49, 50). A systematic review and meta-analysis, including 19 different studies comparing socio-cognitive performance between individuals diagnosed with SSD and those diagnosed with ASD, reported that the level of SC impairment was similar across the disorders: no significant differences emerged in ToM tasks, emotional intelligence and social skills, and, although patients with SSD had a better performance in emotion perception, only a modest effect size was observed (51). These results were however limited by a significant heterogeneity in the tasks employed in the individual studies and by the small sample sizes. A more recent study has therefore performed a comprehensive evaluation of SC performance in large samples of adult subjects with schizophrenia, ASD and typical development, confirming that the level of impairment is very similar between the two disorders, with small differences that become non-significant when the analyses are controlled for symptoms severity (52). These results could suggest that interventions which have shown effectiveness in improving SC performance in one condition could lead to positive results if adopted in the other.

However, as much as the clinical observation and measurement of this overlap between the spectra is important and interesting, a deeper understanding of the neurobiological and molecular mechanisms underlying SC deficits of both disorders, with particular attention to which aspects are shared and which are divergent in the two conditions, could represent a relevant improvement in the perspective of developing and implementing dedicated treatment strategies. In fact, deficits in social interactions and in SC performance observed in SSD and ASD could result either from similar, partially overlapping, independent or even completely opposite neurobiological causes: the latter case has been observed in different independent studies, leading to the hypothesis that SSD and ASD may represent diametrically divergent disorders of the social brain (53, 54). Moreover, as SSD and ASD share a number predisposing neurodevelopmental features and risk factors, it has been theorized that the co-occurrence of the two disorders, or of different neurobiological alterations belonging to the two spectra, could be frequent, explaining the association and similarities often observed on a clinical level (43).

### Social Cognition as a Research Domain Criteria

The RDoC project represents a framework for research that conceptualizes mental illnesses as brain disorders and assumes that the dysfunctions in neural circuits can be identified with the tools of clinical neuroscience and genetics. Data obtained in this perspective could yield specific biosignatures, possibly leading to an improvement in the clinical management of psychiatric disorders (55).

Disassembling the traditional diagnostic categories established in psychiatry on the basis of clinical observation does not represent the aim of the RDoC approach; rather, among its primary goals features a deeper understanding of neural circuits’ functioning that could result in better knowledge of the causal relationships beyond symptoms and behaviors occurring in different disorders (56).

This might also represent a step forward in meeting the need of a more personalized medicine in psychiatry by improving the characterization of individual cases, an objective that is somehow currently difficult with the information conveyed by the diagnosis alone (57); this approach, including direct comparisons of clinical disorders, is recently attracting more scientific attention (58).

In particular, the RDoC perspective could be interesting in the study of neurodevelopmental disorders, as the developmental trajectory of different conditions currently represents an important object on neuroanatomical, neurobiological, molecular and behavioral research (59).

SC has been proposed as a major RDoC domain on the basis of the neurobiological evidences defining the brains systems involved in socio-cognitive processes and for its relevance as a transdiagnostic clinical construct (60).

Several studies have been performed to date to elucidate the roles played in SC by specific neural structures, genes, and neurotransmitter systems (61–64).

### Neuroanatomical and Neurofunctional Brain Markers of Social Cognition

SC involves a broad range of neural regions and networks in stimulus processing in the central nervous system. Neuroimaging studies represent an important tool for the comprehension of the neural bases that explain the mechanisms of SC, since they can provide not only an assessment of brain anatomy but also of neural activity in specific regions as well as its relations (65–69). In this sense, the use of structural Magnetic Resonance Imaging (sMRI) and functional Magnetic Resonance Imaging (fMRI) has become a fundamental strategy for understanding these neural bases, as well as for studying psychiatric diseases that classically present alterations in SC, such as ASD and SSD (70–73). Several neuroimaging studies have identified specific brain areas most frequently involved in SC, but also networks formed by the connections between these focal brain areas (70, 74). This group of brain regions may also be collectively referred to as the “social brain” (75, 76). It comprises the following areas, all relevant in SC processes: the prefrontal cortex (PFC) and its subdivisions, that are dorsomedial, dorsolateral, ventromedial, ventrolateral and orbitofrontal, the amygdala, the thalamus, the anterior cingulate cortex (ACC), the posterior cingulate cortex (PCC), the temporal cortex more specifically the surroundings of the superior temporal sulcus (STS) and temporo-parietal junction (TPJ), and occipitotemporal regions, encompassing the fusiform gyrus (70, 75). Other regions that are also involved in the SC phenotype are the somatosensory areas and motor cortex (71, 72). Although many studies of SC impairments in ASD and SSD show alterations in most of the aforementioned brain regions (77–79), there is an increasing consensus that the abnormalities are usually not focal, but are rather distributed in functional brain networks important to support social functions (71, 74, 75, 80).

#### Prefrontal Cortex

Classically, research studies have focused in structural and functional changes in specific brain areas related to the SC process to describe the neural bases of ASD and SSD (77–79). Indeed, focal alterations in the PFC are largely described in ASD and SSD. Specifically, the medial PFC is recruited in tasks that need conscious attribution or judgment of mental states, traits or dispositional intentions of the individual or others. This region is also involved in the interpretation of non-verbal social information and in the contextual interpretation of complex social information, such as inferring the beliefs of others (81). Activation of the medial PFC is also involved in emotion generation, especially when assessing self-relevant characteristics or emotional awareness (82, 83). The ventrolateral PFC is involved in adaptive responses to social situations, modulating the influence of emotional stimuli on cognition in relation to socially appropriate behaviors (84, 85). A meta-analysis of studies using fMRI in SC tasks, directly comparing patients with SSD and ASD, pointed to important results (79). In this study both groups showed hypoactivation in the medial PFC during ToM related tasks, more pronounced in ASD patients. On the other hand, ventrolateral PFC disruption in facial emotion recognition (FER) tasks was associated mostly with SSD. The finding of reduced ventrolateral PFC, implying connection to social appropriateness of behavior, may be more relevant to SSD patients, while in both disorders, reduced medial PFC activation may contribute to alterations in conscious awareness of others’ emotional states (79). Further studies using fMRI in social tasks have demonstrated heterogeneous results, with either hypoactivation in the medial PFC in patients with ASD (86) and SSD (87) and a hyperactivation of the PFC in patients with SSD (88) and ASD (89). The involvement of the PFC in SC impairment has also been demonstrated in morphometric studies, suggesting a reduction in the PFC gray matter volume in patients with ASD (78, 89) and in those with SSD (90).

#### Amygdala

Besides frontal regions, the amygdala structure also contributes to SC by mediating arousal or biological salience associated with different stimuli (91). This structure is also involved in recognizing facial emotional expressions and in evaluating stimuli (72). Both SSD and ASD patients present amygdala hypoactivation when processing social stimuli and this may occur in a stimulus type dependent manner, with SSD patients presenting alteration in tasks related to the attribution of affective states (FER) and ASD individuals showing impairment in tasks related to epistemic and intentional attributions (ToM) (79). These findings seem to be particularly related to the known deficits in emotion perception among persons with ASD (92). Corroborating the involvement of the amygdala in the social cognitive dysfunctions in these two disorders, other studies have demonstrated that this structure present both volumetric changes in ASD and SSD (78, 93, 94) as well as functional alterations in ASD (76, 89). The amygdala of toddlers and children with ASD was reported to be significantly enlarged relative to controls and this increase in amygdala volume was accompanied by more severe impairments in the social and communication aspects (94). On the other hand, patients with ASD showed smaller gray matter volume in the amygdala compared to controls (78) and the amygdala volume was also found to be smaller in SSD patients, compared to controls (93). As for functional alterations, a meta-analysis revealed differences in activation in the amygdala between ASD and typically developing individuals,with ASD showing reduced activity in amygdala in face processing tasks (89). Lower level of amygdala activation has been also found to play a important role in social and emotional processing in ASD (95, 96). Also, the amygdala showed reduced activity in ASD group compared with the typically developing group in the processing of emotional facial expressions (76).

#### Thalamus

Another structure also involved in SC is the thalamus, which plays a role in coordinating the information flow in various cognitive and sensory processes (97). The thalamus is directly involved in visual perception (directing attention towards salient stimuli) and its dorsomedial portion is associated with executive functions through its connections with the PFC. Atrophy and impaired function were observed in the thalamus of ASD subjects from late childhood to adulthood (98). In ASD individuals, a decrease in the right thalamus volume after a developmental period of two years was reported, and it was correlated with social deficits, while typically developing controls did not show volume change in this structure (99). Also, abnormalities of verbal and nonverbal communication in ASD individuals are probably due to thalamic hyperactivation and subsequent dysfunction of other areas such as visual cortex and frontal regions (98). In SSD patients, there exist consistent evidence for structural changes (both reduced volume and cell numbers) in the pulvinar located in the posterior thalamus and also evidence that the thalamo-cortical dysfunction in this disorder might be attributable to structural alterations in the thalamus (100). In SSD patients a decreased engagement of the thalamus during SC tasks in comparison to controls was observed (79). Another study suggested that changes in thalamic activation appear to play a fundamental role in the development of both ASD and SSD (98).

#### Cingulate Cortex

The regions surrounding the cingulate cortex are also referred to as important areas in the evaluation of SC (76, 79, 93, 94). The ACC is associated with processing positive and negative judgments of social situations and integrating such judgments with emotional information to motivate behavior patterns (72). The PCC is associated with mentalizing or inferring others’ mental states (70). ASD subjects showed more engagement of the ACC and PCC in comparison to SSD in FER tests. SSD patients showed greater engagement in PCC in comparison to ASD individuals in ToM tasks (79). Another previous meta-analysis comparing grey matter deficits in ASD and SSD had reported common deficits in right PCC (93). A meta-analysis showed a positive relationship between ACC gray matter thinning and high risk for SSD, which may be associated with increasing social withdrawal (101), while for ASD individuals the alterations in the regions of the cingulate cortex appears to be more functional than structural (102). The findings of altered recruitment of cingulate cortex in ASD was also reported by other authors (76, 89, 94).

#### Somatosensory Cortices

Social interactions also involve the somatosensory cortices, as these brain areas play a role in internal representations of affective states. Engagement of somatosensory cortices is related to invoking mirror bodily states associated with relevant emotions or other internal states and facilitates their recognition in oneself or in others (103). In ASD patients, there was a hypoactivation in somatosensory cortices, in comparison to controls and to SSD patients, during both FER and ToM tasks, which corroborates the dysfunction in invoking mirroring mechanisms when processing social stimuli observed in ASD. On the other hand, the increased engagement of somatosensory regions in SSD individuals may explain hyper-mentalization states that can be found in these patients (79). Subsequent findings also pointed to changes in the somatosensory cortex of patients with SSD and ASD, with both groups presenting weaker cortical responses to visual, somatosensory and auditory stimuli in sensory fMRI, compared to controls. However, in ASD individuals there were greater cortical variabilities, whereas in SSD patients there were smaller response amplitudes (104). All these findings might help differentiate between the two groups and aid in the elucidation of neural diverse mechanisms underlying each disorder.

#### Temporal Lobe Regions

Finally, temporal lobe regions, including the STS and TPJ, are also key components of the social brain. The regions around STS play a major role in social perception by analyzing biological motion cues, including gaze direction, body movements and facial expressions. This is important in inferring or formulating attributions about others’ intentional or affective states (105). The involvement of the STS in the SC of patients with ASD and SSD are demonstrated by numerous studies, often reporting heterogeneous results (79, 87, 89, 106). For example, similar hypoactivation in these regions during ToM tasks were found in SSD and ASD. On the other hand, ASD patients showed increased engagement in these regions in comparison to controls and to SSD patients during FER tasks (79). Both ASD and SSD showed hypoactivation in the STS, compared to controls, for the contrast intentional versus physical information processing. Relative increased activation for physical information processing in SSD and relative decreased activation for intentional information processing in ASD patients were observed, endorsing differences between the groups (87). The TPJ have been linked with SC tasks requiring individuals to ‘‘think about other people’s thoughts’’ or to take another perspective about affective or cognitive states of others (107, 108). The TPJ was hypoactivated in ToM tasks in ASD patients, in comparison to controls. The TPJ was more activated in FER tasks in SSD patients, in comparison to ASD individuals (79). The involvement of TPJ in the SC process has also been demonstrated in more recent studies in patients with SSD (88, 109) and ASD (89).

#### Neural Networks

More recently, there has been a major interest in broadening the study of structural alterations underlying the neural basis of these two disorders to include the relationship between the functionality of different brain regions, combined in more complex and connected neural networks. In this sense, when expanding the scope from changes in focal brain areas to include broader alterations in neural functional networks, a new range of possibilities is found, opening the door to wider explanations for the common neural bases among ASD and SSD, as well as for their differences (71, 74, 75, 80, 110).

The connection between areas belonging to the frontal lobe and the temporal lobe are commonly described in studies assessing SC in ASD and SSD. Changes in connectivity patterns between the STS and frontal regions in SC processes have been demonstrated in patients with ASD and SSD (87, 106, 111), as well as in patients diagnosed with early psychosis (112). Still regarding frontotemporal connections, Eack and collaborators found increased frontotemporal and orbito-frontal connectivity in ASD patients and decreased connectivity between the same areas in SSD patients (71). A recent study also described connections between frontotemporal areas through fMRI evaluation of the ToM network (which is composed of connections between medial PFC, STS, TPJ and precuneus) in patients with SSD, revealing a raise in these connectivities during emotional peaks in comparison to controls (113).

Networks involving connections in fronto-parietal areas also seem to play an important role in the SC of patients with ASD and SSD. A study using a connectivity approach (74) found that, although both groups present significant enrichment in the frontoparietal and limbic networks regarding the cortical thickness of structures involved in these networks, this occurs in opposite directions, with SSD patients showing increased cortical thickness and those with ASD presenting decreased cortical thickness.

Regarding the surface area, patients with ASD present increased surface values of structures involved in the ventral attention network, while those with SSD present decreased values (74). The ventral attention network involves the TPJ and the ventral frontal cortex and is usually recruited when behaviorally relevant stimuli occur unexpectedly (for instance, when they appear outside the focus of spatial attention) (114).

Other neural networks described as possible protagonists in the SC process in ASD and SSD are the Default Mode Network (DMN) and the Salience Network (SN). The DMN is a major network encompassing the medial PFC, PCC, precuneus and bilateral inferior parietal lobules, which is activated when there is no engagement in any specific task and deactivated in the context of effortful cognitive tasks and SC tasks (115–118). The SN is a task activated brain network and comprises the anterior insula, dorsal ACC, the anterior PFC and the thalamus (119). This network is related to redirecting attention to unexpected but salient stimuli and is involved in SC, non-SC and emotional processes (120). A recent study showed distinct atypical connections in the DMN and SN in ASD and SSD patients, with ASD individuals showing altered intra-SN connections and SSD participants showing inter-DMN-SN atypical connections (80). These findings may suggest that, although ASD and SSD have common neural networks with regard to changes in SC, these two conditions may differ in the way in which these networks are involved.

All the aforementioned findings suggest that the assessment of the neural bases involved in these psychiatric diseases should also be analyzed through coordinated and diffuse changes in networks responsible for the processing of complex human traits and not just through focal structural changes.

### Molecular Biomarkers of Social Cognition

To date, most studies carried out on molecular mechanisms of SC have been focused on neuropeptides oxytocin and arginine vasopressin receptors (*OXTR* and *AVPR*, respectively) genes, since the OXT and AVP neuropeptides have been largely involved in a wide range of social behaviors (121). OXT is a key modulator of the most intuitive and yet most complex socioemotional behaviors. OXT affects social cognition by enhancing the salience of social cues and reward sensitivity to these cues (122). OXT is associated to various forms of social attachments and affects the activity and the connectivity of a social brain network that includes the areas described above.

In humans, the *OXTR* gene is located on chromosome 3p25.3, and one of the most studied single nucleotide polymorphism (SNP) is the rs53576, which consists of a guanine (G) to adenine (A) change within the third intron of *OXTR*. This SNP has been associated with SC phenotypes, such as empathy (123–127), prosocial behaviors (128, 129), and social abilities (130). Although some contrasting results exist, evidence for the different phenotypes related to SC converge in demonstrating a deficit of A allele carriers that showed less dispositional empathy (124) and lower trust behavior (128). Moreover, several studies have been shown that the rs53576 A allele represents a genetic risk for SC, because social dysfunctions of A allele subjects was reflected in morphometric alterations of the hypothalamus and amygdala, as well as on the structural connectivity of the system limbic structures involved in social behaviors (123, 129). *OXTR* SNPs rs7632287 and rs2254298, are yet other interesting polymorphisms whose associations with SC phenotypes were reported (126, 131–134). Although all these studies indicate that there is an association between *OXTR* gene and SC dysfunctions, previous studies neither specify the nature of associations found in an unequivocal way nor select genotypes that are the basis for this association. Moreover, contrasting results have been published about correlation of peripheral plasma concentrations of oxytocin with *OXTR* SNPs as well as central nervous system level of this peptide (135, 136).

Interestingly, variability in *OXTR* methylation has been associated with differences in SC and brain response during social tasks (137, 138). In particular, DNA methylation of *OXTR* has been shown to negatively correlate with *OXTR* transcription across tissues indicating that increased levels of methylation correspond to greater deficits in social responsiveness (139, 140) in ASD subjects.

Finally, few studies with contrasting results are performed to date concerning correlation between oxytocin plasma levels and social cognition (141, 142). In the case of AVP, the genes encoding the 3 receptors (*AVPR1a*, *AVPR1b*, and *AVPR2*) and are located on chromosomes 12q14, 1q32, and Xq28, respectively. Concerning the relationship with SC, the most studied SNPs are SSRs, such as RS3 and RS1 located in the *AVPR1* gene, that along with others, were investigated in association to SC phenotypes with sparse and contrasting results (126, 130, 134, 143). In the same way, in literature few studies with conflicting data are present regarding correlations between blood AVP concentrations (141, 144) and SC phenotypes. Indeed, literature on SNPs in the *AVPR* genes is extremely poor, and therefore, further research is required to confirm or reject the hypothesis of their association with SC dysfunctions, in ASD as well as in SSD.

To date, in addition to *OXTR* and *AVPR* SNPs, sparse results on other systems were reported in correlation with SC in humans. Emotional behaviors are accompanied by biochemical changes *via* dopamine catabolism. The catechol-O-methyltransferase (*COMT*) is the major enzyme responsible for degrading amines like dopamine, norepinephrine, and epinephrine. The most studied polymorphism is the Val158Met *COMT* functional SNP that has been associated with differential response to affect in prefrontal brain areas and limbic structures and for this reason widely investigated in association with SC. In healthy volunteers, carriers of the Val allele that have an enhanced *COMT* enzyme activity compared to Met/Met-allele carriers, showed an increase in social cooperative behavior and a stronger response to social interactions (145). Moreover, Val homozygotes were more altruistic, empathetic, and cooperative than Met homozygotes (146). On the other hand, regarding SC, it has been suggested that Met allele confers more intensive emotional processing, with more anxiety and sensitive behavior in response to aversive stimuli, as well as habitually experienced more negative affect and negative attentional bias (146–149). However, to date the effect of the *COMT* gene on SC has not been sufficiently investigated in SSD and in ASD. Indeed, to date few, spare and contrasting results are available since *COMT* gene was investigated primarily in SSD and negative associations often has been provided (150, 151).

Some other candidate genes as well as immune markers were investigated in association to SC mainly in SSD, such as anti-inflammatory cytokine IL-10 that was associated with ToM, but no further strong replication occurred (152–155).

Finally, several studies reported associations between genetics and SC also in subjects with the 22q11.2 Deletion Syndrome (22q11.2 DS) that has a robust representation of genetic proneness to SSD (156–161). There is a strong agreement in all the results reported showing that compared to healthy controls, 22q11.2 CNV subjects showed significantly poorer SC such as emotion differentiation, emotion recognition, lie detection, sarcasm detection.

## Discussion

In a neuroanatomical and neurofunctional perspective, the regions interested in SC have recently been the focus of a growing body of evidence. The brain areas that appear to be altered in relation to deficits of SC are largely shared in SSD and ASD; however, the results of various studies suggest that, in some cases, the qualitative nature of these alterations may be different in the two spectra. In particular, some relevant differences could be present at the level of brain networks and connections (71, 80) (**Table 1**).

**Table 1:** Neurobiological features involving social cognition in SSD and ASD.

	Neuroanatomical and Neurofunctional Features	Neural Connections and Networks
**Similar** **Alterations**	Amygdala: hypoactivation when processing social stimuli. Thalamus: reduced volume and dysfunctions in SC tasks.	None
**Different Alterations**	SSC: hypoactivation in ASD and hyperactivation in SSD in SC tasks.	OF connections: increased in ASD and decreased in SSD. FP connections: decreased CT in the connected areas in ASD and increased CT in SSD. VAN: increased surface values in the involved structures in ASD and decreased surface values in SSD. DMN-SN: altered intra SN connections in ASD and altered inter DMN-SN connections in SSD.
**Inconclusive or Conflicting Literature**	ACC; PCC; PFC; STS; TPJ.	FT connections

ACC, anterior cingulate cortex; ASD, autism spectrum disorder; CT, cortical thickness; DMN, Default Mode Network; FP, frontoparietal; FT, frontotemporal; OF, orbitofrontal; PCC, posterior cingulate cortex; PFC, prefrontal cortex; SC, social cognition; SN, Salience Network; SSC, somatosensory cortex; SSD, schizophrenia spectrum disorder; STS, superior temporal sulcus; TPJ, Temporo-Parietal Junction; VAN, Ventral Attentive Networks.

Although on a clinical level SC deficits in SSD and ASD appear to largely superimposable, suggesting that interventions that are effective in one spectrum could also be adopted in the other (52), further exploring the commonalities and the potential differences on a neurobiological level could provide additional confirmations to this hypothesis, but also lead to the development of specific and targeted treatments. Moreover, investigating with neuroimaging tools subjects diagnosed with SSD and showing prominent ASD features (39), and those diagnosed with ASD with relevant psychotic symptoms (42, 43), and evaluating if the neuroanatomical and neurofunctional profile of these individuals represents an intermediate phenotype or not, could provide further insight and represent an interesting perspective for future studies.

The neuroimaging findings related to SC in SSD and ASD, if considered in an RDoC context, confirm the importance of developing a framework based on neurobiological phenotypes and malfunctions, as the diagnostic categories currently employed in psychiatric practice do not appear to properly represent distinct nosological entities. This could be especially true when considering disorders as clinically heterogeneous and complex as SSD and ASD, which may present important areas of overlap, but may also present relevant interindividual differences even within each of the two spectra.

On the contrary, literature concerning molecular markers related to SC is scarce and mainly focused on candidate gene studies, and potential commonalities and divergences between SSD and ASD on a molecular level still have to be further investigated. SC is a highly complex process requiring a vast regulatory network involving genetic, epigenetic, and environmental factors, consequently the use of powerful tools, such as genome-wide association studies (GWAS) is needed. Moreover, functional and structural brain imaging studies could also help in understanding the role of genetic variants in the development of SC phenotypes (159, 162). This link between genetics and neuroimaging changes can be explained, among other factors, by the role that genes have in regulating both synaptogenesis, synaptic function and the formation of neuronal circuits (75). Indeed, the combination of genetics and neuroimaging in a study of the association between variants of genetic loci linked to SSD and SC in healthy individuals found that those with an increased risk score (taking into account the combined risk of such genetic loci) presented changes in the ACC when evaluating episodic memory and changes in the PCC when the ToM was evaluated (162).

Some inconsistencies across studies were observed both in neuroimaging correlates and behavioral performance of SC in SSD and ASD, which can partly be the results of differences in task design: task characteristics have been shown to have an influence on outcomes and interpretation of social cognition performance assessment, and the choice of appropriate measures, balancing task sensitivity and ecological validity, represents an important factor that should be consistently taken account in the design of future studies (163). Moreover, it is possible that isolating SC in different components, such as emotion processing and ToM, might not be ideal, as in the real-world context of interpersonal relationships all these separate domains are likely to be involved in determining social behaviors (164).

The selection of evidences presented and discussed in the present review was not based on a systematic literature research, therefore the possibility that some study of potential interest may not have been included represents a limitation. However, the aim of this work was to provide a narrative and critical overview of current evidences highlighting the interest of implementing a RDoC approach in the study of SC in SSD and ASD, and the development of a systematic and comprehensive review investigating this topic represents a valid perspective for future research.

Research on neurobiological and molecular mechanisms underlying socio-cognitive functioning is an expanding field of notable scientific interest and is providing valuable insight in understanding the overlaps between SSD and ASD. However, more research is currently needed to define specific endophenotypes that could benefit from targeted treatments and interventions, concretely fulfilling the objectives that the RDoC project proposes as essential goals (55, 60). Indeed, there is some evidence of oxytocin’s modulation of SC brain functions with intranasal oxytocin (165), however, contrasting results have been published about this issue, and although intranasal oxytocin seems to have potential therapeutic value, there are key questions that remain unanswered as to decide the optimal target groups and treatment course (166).

## Conclusions

Current studies on neuroanatomical and neurofunctional bases of SC deficits are providing valuable insights in the overlaps and differences between SSD and ASD. However, more research is required in this field, in particular regarding molecular and genetic aspects. Applying the RDoC approach to further the study of SC in SSD and ASD could lead to a considerable improvement in the understanding of both spectra, with potential positive repercussion in the perspective of implementing these findings in clinical practice.

## Author Contributions

GN, SB, AM, and RCS participated in the writing process of the first draft of the manuscript. AC and GD made literature search and independently reviewed electronic databases. AV, CT, SB, and AM revised the final version of the manuscript. All authors contributed to the article and approved the submitted version.

## Conflict of Interest

The authors declare that the research was conducted in the absence of any commercial or financial relationships that could be construed as a potential conflict of interest.

## References

[1] Nuechterlein KH Green MF Kern RS Baade LE Barch DM Cohen JD The MATRICS Consensus Cognitive Battery, part 1: test selection, reliability, and validity. Am J Psychiatry (2008) 165:203–13. 10.1176/appi.ajp.2007.07010042 18172019

[2] Penn DL Sanna LJ Roberts DL Social Cognition in Schizophrenia: An Overview. Schizophr Bull (2008) 34:408–11. 10.1093/schbul/sbn014 PMC2632430 18375928

[3] Pinkham AE Penn DL Green MF Buck B Healey K Harvey PD The social cognition psychometric evaluation study: results of the expert survey and RAND panel. Schizophr Bull (2014) 40:813–23. 10.1093/schbul/sbt081 PMC4059426 23728248

[4] Galderisi S Rucci P Kirkpatrick B Mucci A Gibertoni D Rocca P Interplay Among Psychopathologic Variables, Personal Resources, Context-Related Factors, and Real-life Functioning in Individuals With Schizophrenia: A Network Analysis. JAMA Psychiatry (2018) 75:396–404. 10.1001/jamapsychiatry.2017.4607 29450447 PMC5875306

[5] Galderisi S Rossi A Rocca P Bertolino A Mucci A Bucci P The influence of illness-related variables, personal resources and context-related factors on real-life functioning of people with schizophrenia. World Psychiatry (2014) 13:275–87. 10.1002/wps.20167 PMC4219069 25273301

[6] Green MF Horan WP Lee J Nonsocial and social cognition in schizophrenia: current evidence and future directions. World Psychiatry (2019) 18:146–61. 10.1002/wps.20624 PMC6502429 31059632

[7] Green MF Horan WP Lee J Social cognition in schizophrenia. Nat Rev Neurosci (2015) 16:620–31. 10.1038/nrn4005 26373471

[8] Harvey PD Penn D Social cognition: the key factor predicting social outcome in people with schizophrenia? Psychiatry Edgmont Pa Townsh (2010) 7:41–4. PMC2848464 20376275

[9] Harvey PD Strassnig M Predicting the severity of everyday functional disability in people with schizophrenia: cognitive deficits, functional capacity, symptoms, and health status. World Psychiatry (2012) 11:73–9. 10.1016/j.wpsyc.2012.05.004 PMC3363376 22654932

[10] Mancuso F Horan WP Kern RS Green MF Social Cognition in Psychosis: Multidimensional Structure, Clinical Correlates, and Relationship With Functional Outcome. Schizophr Res (2011) 125:143–51. 10.1016/j.schres.2010.11.007 PMC3073542 21112743

[11] Pinkham AE Harvey PD Penn DL Social Cognition Psychometric Evaluation: Results of the Final Validation Study. Schizophr Bull (2018) 44:737–48. 10.1093/schbul/sbx117 PMC6007629 28981848

[12] Pinkham AE Penn DL Green MF Harvey PD Social Cognition Psychometric Evaluation: Results of the Initial Psychometric Study. Schizophr Bull (2016) 42:494–504. 10.1093/schbul/sbv056 25943125 PMC4753585

[13] Chung YS Barch D Strube M A meta-analysis of mentalizing impairments in adults with schizophrenia and autism spectrum disorder. Schizophr Bull (2014) 40:602–16. 10.1093/schbul/sbt048 PMC3984506 23686020

[14] Green MF Bearden CE Cannon TD Fiske AP Hellemann GS Horan WP Social Cognition in Schizophrenia, Part 1: Performance Across Phase of Illness. Schizophr Bull (2012) 38:854–64. 10.1093/schbul/sbq171 PMC3406534 21345917

[15] Ludwig KA Pinkham AE Harvey PD Kelsven S Penn DL Social Cognition Psychometric Evaluation (SCOPE) in People with Early Psychosis: A Preliminary Study. Schizophr Res (2017) 190:136–43. 10.1016/j.schres.2017.03.001 PMC5735418 28302395

[16] Phillips LK Seidman LJ Emotion Processing in Persons at Risk for Schizophrenia. Schizophr Bull (2008) 34:888–903. 10.1093/schbul/sbn085 18644853 PMC2518637

[17] Lai M-C Lombardo MV Baron-Cohen S Autism. Lancet Lond Engl (2014) 383:896–910. 10.1016/S0140-6736(13)61539-1 24074734

[18] Velikonja T Fett A-K Velthorst E Patterns of Nonsocial and Social Cognitive Functioning in Adults With Autism Spectrum Disorder: A Systematic Review and Meta-analysis. JAMA Psychiatry (2019) 76:135–51. 10.1001/jamapsychiatry.2018.3645 PMC6439743 30601878

[19] Howlin P Moss P Savage S Rutter M Social outcomes in mid- to later adulthood among individuals diagnosed with autism and average nonverbal IQ as children. J Am Acad Child Adolesc Psychiatry (2013) 52:572–81. 10.1016/j.jaac.2013.02.017 23702446

[20] Joshi G Wozniak J Petty C Martelon MK Fried R Bolfek A Psychiatric comorbidity and functioning in a clinically referred population of adults with autism spectrum disorders: a comparative study. J Autism Dev Disord (2013) 43:1314–25. 10.1007/s10803-012-1679-5 23076506

[21] Orsmond GI Shattuck PT Cooper BP Sterzing PR Anderson KA Social participation among young adults with an autism spectrum disorder. J Autism Dev Disord (2013) 43:2710–9. 10.1007/s10803-013-1833-8 PMC3795788 23615687

[22] Billstedt E Gillberg IC Gillberg C Aspects of quality of life in adults diagnosed with autism in childhood: A population-based study. Autism (2011) 15:7–20. 10.1177/1362361309346066 20923888

[23] Mazurek MO Loneliness, friendship, and well-being in adults with autism spectrum disorders. Autism Int J Res Pract (2014) 18:223–32. 10.1177/1362361312474121 24092838

[24] van Heijst BF Geurts HM Quality of life in autism across the lifespan: A meta-analysis. Autism (2015) 19:158–67. 10.1177/1362361313517053 24443331

[25] Sasson NJ Morrison KE Kelsven S Pinkham AE Social cognition as a predictor of functional and social skills in autistic adults without intellectual disability. Autism Res (2020) 13:259–70. 10.1002/aur.2195 31419075

[26] American Psychiatric Association Diagnostic and Statistical Manual of Mental Disorders (DSM-5®). American Psychiatric Association: Arlington, VA (2013).

[27] Barlati S Deste G Ariu C Vita A Autism Spectrum Disorder and Schizophrenia: Do They Overlap? Int J Emerg Ment Health Hum Resil (2016) 18:760–3. 10.4172/1522-4821.1000318

[28] Dell’Osso L Luche RD Maj M Adult autism spectrum as a transnosographic dimension. CNS Spectr (2016) 21:131–3. 10.1017/S1092852915000450 26349624

[29] Hommer RE Swedo SE Schizophrenia and Autism—Related Disorders. Schizophr Bull (2015) 41:313–4. 10.1093/schbul/sbu188 PMC4332956 25634913

[30] King BH Lord C Is schizophrenia on the autism spectrum? Brain Res (2011) 1380:34–41. 10.1016/j.brainres.2010.11.031 21078305

[31] Sasson NJ Pinkham AE Carpenter KLH Belger A The benefit of directly comparing autism and schizophrenia for revealing mechanisms of social cognitive impairment. J Neurodev Disord (2011) 3:87–100. 10.1007/s11689-010-9068-x 21484194 PMC3188289

[32] Vannucchi G Masi G Toni C Dell’Osso L Marazziti D Perugi G Clinical features, developmental course, and psychiatric comorbidity of adult autism spectrum disorders. CNS Spectr (2014) 19:157–64. 10.1017/S1092852913000941 24352005

[33] Crescenzo FD Postorino V Siracusano M Riccioni A Armando M Curatolo P Autistic Symptoms in Schizophrenia Spectrum Disorders: A Systematic Review and Meta-Analysis. Front Psychiatry (2019) 10:78. 10.3389/fpsyt.2019.00078 30846948 PMC6393379

[34] Kincaid DL Doris M Shannon C Mulholland C What is the prevalence of autism spectrum disorder and ASD traits in psychosis? A systematic review. Psychiatry Res (2017) 250:99–105. 10.1016/j.psychres.2017.01.017 28152400

[35] Deste G Vita A Penn DL Pinkham AE Nibbio G Harvey PD Autistic symptoms predict social cognitive performance in patients with schizophrenia. Schizophr Res (2020) 215:113–9. 10.1016/j.schres.2019.11.008 PMC7035981 31780344

[36] Ziermans TB Schirmbeck F Oosterwijk F Geurts HM de Haan L Outcome of Psychosis (GROUP) Investigators Autistic traits in psychotic disorders: prevalence, familial risk, and impact on social functioning. Psychol Med (2020) 1–10. 10.1017/S0033291720000458 PMC8327624 32151297

[37] Deste G Vita A Nibbio G Penn DL Pinkham AE Harvey PD Autistic symptoms and social cognition predict real-world outcomes in patients with schizophrenia. Front Psychiatry (2020) 11:524. 10.3389/fpsyt.2020.00524 32581892 PMC7294984

[38] Harvey PD Deckler E Jones MT Jarskog LF Penn DL Pinkham AE Autism symptoms, depression, and active social avoidance in schizophrenia: Association with self-reports and informant assessments of everyday functioning. J Psychiatr Res (2019) 115:36–42. 10.1016/j.jpsychires.2019.05.010 31102902 PMC6556410

[39] Barlati S Deste G Gregorelli M Vita A Autistic traits in a sample of adult patients with schizophrenia: prevalence and correlates. Psychol Med (2019) 49:140–8. 10.1017/S0033291718000600 29554995

[40] Deste G Barlati S Gregorelli M Lisoni J Turrina C Valsecchi P Looking through autistic features in schizophrenia using the PANSS Autism Severity Score (PAUSS). Psychiatry Res (2018) 270:764–8. 10.1016/j.psychres.2018.10.074 30551322

[41] Downs JM Lechler S Dean H Sears N Patel R Shetty H The association between co-morbid autism spectrum disorders and antipsychotic treatment failure in early-onset psychosis: a historical cohort study using electronic health records. J Clin Psychiatry (2017) 78:e1233–41. 10.4088/JCP.16m11422 PMC6037287 29125721

[42] Chandrasekhar T Copeland JN Spanos M Sikich L Autism, Psychosis, or Both? Unraveling Complex Patient Presentations. Child Adolesc Psychiatr Clin (2020) 29:103–13. 10.1016/j.chc.2019.08.003 31708040

[43] Chisholm K Lin A Abu-Akel A Wood SJ The association between autism and schizophrenia spectrum disorders: A review of eight alternate models of co-occurrence. Neurosci Biobehav Rev (2015) 55:173–83. 10.1016/j.neubiorev.2015.04.012 25956249

[44] Sullivan S Rai D Golding J Zammit S Steer C The Association Between Autism Spectrum Disorder and Psychotic Experiences in the Avon Longitudinal Study of Parents and Children (ALSPAC) Birth Cohort. J Am Acad Child Adolesc Psychiatry (2013) 52:806–14.e2. 10.1016/j.jaac.2013.05.010 23880491

[45] Selten J-P Lundberg M Rai D Magnusson C Risks for Nonaffective Psychotic Disorder and Bipolar Disorder in Young People With Autism Spectrum Disorder: A Population-Based Study. JAMA Psychiatry (2015) 72:483–9. 10.1001/jamapsychiatry.2014.3059 25806797

[46] Volkmar FR Cohen DJ Comorbid association of autism and schizophrenia. Am J Psychiatry (1991) 148:1705–7. 10.1176/ajp.148.12.1705 1957933

[47] Larson FV Wagner AP Jones PB Tantam D Lai M-C Baron-Cohen S Psychosis in autism: Comparison of the features of both conditions in a dually affected cohort. Br J Psychiatry (2017) 210:269–75. 10.1192/bjp.bp.116.187682 PMC5376719 27979819

[48] Kuo SS Eack SM Meta-Analysis of Cognitive Performance in Neurodevelopmental Disorders during Adulthood: Comparisons between Autism Spectrum Disorder and Schizophrenia on the Wechsler Adult Intelligence Scales. Front Psychiatry (2020) 11:187. 10.3389/fpsyt.2020.00187 32273855 PMC7114889

[49] Bliksted V Ubukata S Koelkebeck K Discriminating autism spectrum disorders from schizophrenia by investigation of mental state attribution on an on-line mentalizing task: A review and meta-analysis. Schizophr Res (2016) 171:16–26. 10.1016/j.schres.2016.01.037 26817402

[50] Gilleen J Xie F Strelchuk D 38. Distinct Theory of Mind Deficit Profiles in Schizophrenia and Autism: A Meta-Analysis of Published Research. Schizophr Bull (2017) 43:S22. 10.1093/schbul/sbx021.057

[51] Fernandes JM Cajão R Lopes R Jerónimo R Barahona-Corrêa JB Social Cognition in Schizophrenia and Autism Spectrum Disorders: A Systematic Review and Meta-Analysis of Direct Comparisons. Front Psychiatry (2018) 9:504. 10.3389/fpsyt.2018.00504 30459645 PMC6232921

[52] Pinkham AE Morrison KE Penn DL Harvey PD Kelsven S Ludwig K Comprehensive comparison of social cognitive performance in autism spectrum disorder and schizophrenia. Psychol Med (2019) 1–9. 10.1017/S0033291719002708 31576783

[53] Crespi B How is quantification of social deficits useful for studying autism and schizophrenia? Psychol Med (2020) 50:523–5. 10.1017/S0033291719003180 31753058

[54] Crespi B Badcock C Psychosis and autism as diametrical disorders of the social brain. Behav Brain Sci (2008) 31:241–61. 10.1017/S0140525X08004214 18578904

[55] Insel T Cuthbert B Garvey M Heinssen R Pine DS Quinn K Research Domain Criteria (RDoC): Toward a New Classification Framework for Research on Mental Disorders. Am J Psychiatry (2010) 167:748–51. 10.1176/appi.ajp.2010.09091379 20595427

[56] Morris SE Cuthbert BN Research Domain Criteria: cognitive systems, neural circuits, and dimensions of behavior. Dialog Clin Neurosci (2012) 14:29–37. 10.31887/DCNS.2012.14.1/smorris PMC3341647 22577302

[57] Maj M Beyond diagnosis in psychiatric practice. Ann Gen Psychiatry (2020) 19:27. 10.1186/s12991-020-00279-2 32322290 PMC7160990

[58] Malcolm A Labuschagne I Castle D Terrett G Rendell PG Rossell SL The relationship between body dysmorphic disorder and obsessive-compulsive disorder: A systematic review of direct comparative studies. Aust N Z J Psychiatry (2018) 52:1030–49. 10.1177/0004867418799925 30238784

[59] Casey BJ Oliveri ME Insel T A Neurodevelopmental Perspective on the Research Domain Criteria (RDoC) Framework. Biol Psychiatry (2014) 76:350–3. 10.1016/j.biopsych.2014.01.006 25103538

[60] Gur RC Gur RE Social cognition as an RDoC domain. Am J Med Genet B Neuropsychiatr Genet (2016) 171:132–41. 10.1002/ajmg.b.32394 PMC4843508 26607670

[61] Adolphs R The neurobiology of social cognition. Curr Opin Neurobiol (2001) 11:231–9. 10.1016/S0959-4388(00)00202-6 11301245

[62] Fernández M Mollinedo-Gajate I Peñagarikano O Neural Circuits for Social Cognition: Implications for Autism. Neuroscience (2018) 370:148–62. 10.1016/j.neuroscience.2017.07.013 28729065

[63] Kimoto S Makinodan M Kishimoto T Neurobiology and treatment of social cognition in schizophrenia: Bridging the bed-bench gap. Neurobiol Dis (2019) 131:104315. 10.1016/j.nbd.2018.10.022 30391541

[64] Porcelli S Van Der Wee N van der Werff S Aghajani M Glennon JC van Heukelum S Social brain, social dysfunction and social withdrawal. Neurosci Biobehav Rev (2019) 97:10–33. 10.1016/j.neubiorev.2018.09.012 30244163

[65] Deuse L Rademacher LM Winkler L Schultz RT Gründer G Lammertz SE Neural correlates of naturalistic social cognition: brain-behavior relationships in healthy adults. Soc Cognit Affect Neurosci (2016) 11:1741–51. 10.1093/scan/nsw094 PMC5091685 27496338

[66] Labek K Viviani R Gizewski ER Verius M Buchheim A Neural Correlates of the Appraisal of Attachment Scenes in Healthy Controls and Social Cognition-An fMRI Study. Front Hum Neurosci (2016) 10:345. 10.3389/fnhum.2016.00345 27458363 PMC4932100

[67] Mukerji CE Lincoln SH Dodell-Feder D Nelson CA Hooker CI Neural correlates of theory-of-mind are associated with variation in children’s everyday social cognition. Soc Cognit Affect Neurosci (2019) 14:579–89. 10.1093/scan/nsz040 PMC6688452 31194250

[68] Tsoi L Dungan J Waytz A Young L Distinct neural patterns of social cognition for cooperation versus competition. NeuroImage (2016) 137:86–96. 10.1016/j.neuroimage.2016.04.069 27165762

[69] Valk SL Bernhardt BC Böckler A Trautwein F-M Kanske P Singer T Socio-Cognitive Phenotypes Differentially Modulate Large-Scale Structural Covariance Networks. Cereb Cortex N Y N 1991 (2017) 27:1358–68. 10.1093/cercor/bhv319 26733538

[70] Arioli M Canessa N Neural processing of social interaction: Coordinate-based meta-analytic evidence from human neuroimaging studies. Hum Brain Mapp (2019) 40:3712–37. 10.1002/hbm.24627 PMC6865826 31077492

[71] Eack SM Wojtalik JA Keshavan MS Minshew NJ Social-cognitive brain function and connectivity during visual perspective-taking in autism and schizophrenia. Schizophr Res (2017) 183:102–9. 10.1016/j.schres.2017.03.009 PMC5432384 28291690

[72] Kozhuharova P Saviola F Ettinger U Allen P Neural correlates of social cognition in populations at risk of psychosis: A systematic review. Neurosci Biobehav Rev (2020) 108:94–111. 10.1016/j.neubiorev.2019.10.010 31730786

[73] Mueller S Keeser D Reiser MF Teipel S Meindl T Functional and structural MR imaging in neuropsychiatric disorders, Part 1: imaging techniques and their application in mild cognitive impairment and Alzheimer disease. AJNR Am J Neuroradiol (2012) 33:1845–50. 10.3174/ajnr.A2799 PMC7964622 22173754

[74] Park MTM Raznahan A Shaw P Gogtay N Lerch JP Chakravarty MM Neuroanatomical phenotypes in mental illness: identifying convergent and divergent cortical phenotypes across autism, ADHD and schizophrenia. J Psychiatry Neurosci JPN (2018) 43:201–12. 10.1503/jpn.170094 PMC5915241 29688876

[75] Müller R-A Fishman I Brain Connectivity and Neuroimaging of Social Networks in Autism. Trends Cognit Sci (2018) 22:1103–16. 10.1016/j.tics.2018.09.008 PMC7080636 30391214

[76] Sato W Uono S The atypical social brain network in autism: advances in structural and functional MRI studies. Curr Opin Neurol (2019) 32:617–21. 10.1097/WCO.0000000000000713 31135458

[77] Pinkham AE Hopfinger JB Pelphrey KA Piven J Penn DL Neural bases for impaired social cognition in schizophrenia and autism spectrum disorders. Schizophr Res (2008) 99:164–75. 10.1016/j.schres.2007.10.024 PMC2740744 18053686

[78] Radeloff D Ciaramidaro A Siniatchkin M Hainz D Schlitt S Weber B Structural alterations of the social brain: a comparison between schizophrenia and autism. PloS One (2014) 9:e106539. 10.1371/journal.pone.0106539 25188200 PMC4154717

[79] Sugranyes G Kyriakopoulos M Corrigall R Taylor E Frangou S Autism spectrum disorders and schizophrenia: meta-analysis of the neural correlates of social cognition. PloS One (2011) 6:e25322. 10.1371/journal.pone.0025322 21998649 PMC3187762

[80] Chen H Uddin LQ Duan X Zheng J Long Z Zhang Y Shared atypical default mode and salience network functional connectivity between autism and schizophrenia. Autism Res (2017) 10:1776–86. 10.1002/aur.1834 PMC5685899 28730732

[81] Amodio DM Frith CD Meeting of minds: the medial frontal cortex and social cognition. Nat Rev Neurosci (2006) 7:268–77. 10.1038/nrn1884 16552413

[82] Johnson SC Baxter LC Wilder LS Pipe JG Heiserman JE Prigatano GP Neural correlates of self-reflection. Brain J Neurol (2002) 125:1808–14. 10.1093/brain/awf181 12135971

[83] Zysset S Huber O Ferstl E von Cramon DY The anterior frontomedian cortex and evaluative judgment: an fMRI study. NeuroImage (2002) 15:983–91. 10.1006/nimg.2001.1008 11906238

[84] Quirk GJ Beer JS Prefrontal involvement in the regulation of emotion: convergence of rat and human studies. Curr Opin Neurobiol (2006) 16:723–7. 10.1016/j.conb.2006.07.004 17084617

[85] Spreng RN Mar RA Kim ASN The common neural basis of autobiographical memory, prospection, navigation, theory of mind, and the default mode: a quantitative meta-analysis. J Cognit Neurosci (2009) 21:489–510. 10.1162/jocn.2008.21029 18510452

[86] Herringshaw AJ Kumar SL Rody KN Kana RK Neural Correlates of Social Perception in Children with Autism: Local versus Global Preferences. Neuroscience (2018) 395:49–59. 10.1016/j.neuroscience.2018.10.044 30419259

[87] Ciaramidaro A Bölte S Schlitt S Hainz D Poustka F Weber B Schizophrenia and autism as contrasting minds: neural evidence for the hypo-hyper-intentionality hypothesis. Schizophr Bull (2015) 41:171–9. 10.1093/schbul/sbu124 PMC4266299 25210055

[88] Fuentes-Claramonte P Martin-Subero M Salgado-Pineda P Santo-Angles A Argila-Plaza I Salavert J Brain imaging correlates of self- and other-reflection in schizophrenia. NeuroImage Clin (2020) 25:102134. 10.1016/j.nicl.2019.102134 31877452 PMC6931228

[89] Patriquin MA DeRamus T Libero LE Laird A Kana RK Neuroanatomical and neurofunctional markers of social cognition in autism spectrum disorder. Hum Brain Mapp (2016) 37:3957–78. 10.1002/hbm.23288 PMC5053857 27329401

[90] Katz J d’Albis M-A Boisgontier J Poupon C Mangin J-F Guevara P Similar white matter but opposite grey matter changes in schizophrenia and high-functioning autism. Acta Psychiatr Scand (2016) 134:31–9. 10.1111/acps.12579 27105136

[91] Santos A Mier D Kirsch P Meyer-Lindenberg A Evidence for a general face salience signal in human amygdala. NeuroImage (2011) 54:3111–6. 10.1016/j.neuroimage.2010.11.024 21081170

[92] Schultz RT Developmental deficits in social perception in autism: the role of the amygdala and fusiform face area. Int J Dev Neurosci (2005) 23:125–41. 10.1016/j.ijdevneu.2004.12.012 15749240

[93] Cheung C Yu K Fung G Leung M Wong C Li Q Autistic disorders and schizophrenia: related or remote? An anatomical likelihood estimation. PloS One (2010) 5:e12233. 10.1371/journal.pone.0012233 20805880 PMC2923607

[94] Ecker C The neuroanatomy of autism spectrum disorder: An overview of structural neuroimaging findings and their translatability to the clinical setting. Autism Int J Res Pract (2017) 21:18–28. 10.1177/1362361315627136 26975670

[95] Kliemann D Rosenblau G Boelte S Heekeren HR Dziobek I Face puzzle—two new video-based tasks for measuring explicit and implicit aspects of facial emotion recognition. Front Psychol (2013) 4:376. 10.3389/fpsyg.2013.00376 23805122 PMC3693509

[96] Zalla T Sperduti M The amygdala and the relevance detection theory of autism: an evolutionary perspective. Front Hum Neurosci (2013) 7:894. 10.3389/fnhum.2013.00894 24416006 PMC3874476

[97] Kumar VJ van Oort E Scheffler K Beckmann CF Grodd W Functional anatomy of the human thalamus at rest. NeuroImage (2017) 147:678–91. 10.1016/j.neuroimage.2016.12.071 28041978

[98] Nakagawa Y Chiba K Involvement of Neuroinflammation during Brain Development in Social Cognitive Deficits in Autism Spectrum Disorder and Schizophrenia. J Pharmacol Exp Ther (2016) 358:504–15. 10.1124/jpet.116.234476 27384073

[99] Zuo C Wang D Tao F Wang Y Changes in the development of subcortical structures in autism spectrum disorder. NeuroReport (2019) 30:1062–7. 10.1097/WNR.0000000000001300 31464839

[100] Dorph-Petersen K-A Lewis DA Postmortem structural studies of the thalamus in schizophrenia. Schizophr Res (2017) 180:28–35. 10.1016/j.schres.2016.08.007 27567291 PMC5746188

[101] Bersani FS Minichino A Fojanesi M Gallo M Maglio G Valeriani G Cingulate Cortex in Schizophrenia: its relation with negative symptoms and psychotic onset. A review study. Eur Rev Med Pharmacol Sci (2014) 18:3354–67. 25491609

[102] Mundy P A review of joint attention and social-cognitive brain systems in typical development and autism spectrum disorder. Eur J Neurosci (2018) 47:497–514. 10.1111/ejn.13720 28922520

[103] Adolphs R Social cognition: feeling voices to recognize emotions. Curr Biol CB (2010) 20:R1071–72. 10.1016/j.cub.2010.11.019 21172624

[104] Haigh SM Gupta A Barb SM Glass SAF Minshew NJ Dinstein I Differential sensory fMRI signatures in autism and schizophrenia: Analysis of amplitude and trial-to-trial variability. Schizophr Res (2016) 175:12–9. 10.1016/j.schres.2016.03.036 PMC4958557 27083780

[105] Grosbras M-H Beaton S Eickhoff SB Brain regions involved in human movement perception: a quantitative voxel-based meta-analysis. Hum Brain Mapp (2012) 33:431–54. 10.1002/hbm.21222 PMC6869986 21391275

[106] Okruszek Ł Wordecha M Jarkiewicz M Kossowski B Lee J Marchewka A Brain correlates of recognition of communicative interactions from biological motion in schizophrenia. Psychol Med (2018) 48:1862–71. 10.1017/S0033291717003385 29173243

[107] Saxe R Kanwisher N People thinking about thinking people. The role of the temporo-parietal junction in “theory of mind.” NeuroImage (2003) 19:1835–42. 10.1016/s1053-8119(03)00230-1 12948738

[108] Saxe R Powell LJ It’s the thought that counts: specific brain regions for one component of theory of mind. Psychol Sci (2006) 17:692–9. 10.1111/j.1467-9280.2006.01768.x 16913952

[109] Briend F Marzloff V Brazo P Lecardeur L Leroux E Razafimandimby A Social cognition in schizophrenia: Validation of an ecological fMRI task. Psychiatry Res Neuroimaging (2019) 286:60–8. 10.1016/j.pscychresns.2019.03.004 30904774

[110] Erdeniz B Serin E İbadi Y Taş C Decreased functional connectivity in schizophrenia: The relationship between social functioning, social cognition and graph theoretical network measures. Psychiatry Res Neuroimaging (2017) 270:22–31. 10.1016/j.pscychresns.2017.09.011 29017061

[111] Hirata K Egashira K Harada K Nakashima M Hirotsu M Isomura S Differences in frontotemporal dysfunction during social and non-social cognition tasks between patients with autism spectrum disorder and schizophrenia. Sci Rep (2018) 8:3014. 10.1038/s41598-018-21379-w 29445197 PMC5813031

[112] Buchy L Barbato M Makowski C Bray S MacMaster FP Deighton S Mapping structural covariance networks of facial emotion recognition in early psychosis: A pilot study. Schizophr Res (2017) 189:146–52. 10.1016/j.schres.2017.01.054 28169088

[113] Hendler T Raz G Shimrit S Jacob Y Lin T Roseman L Social affective context reveals altered network dynamics in schizophrenia patients. Transl Psychiatry (2018) 8:29. 10.1038/s41398-017-0055-9 29382814 PMC5802465

[114] Vossel S Geng JJ Fink GR Dorsal and ventral attention systems: distinct neural circuits but collaborative roles. Neurosci Rev J Bringing Neurobiol Neurol Psychiatry (2014) 20:150–9. 10.1177/1073858413494269 PMC4107817 23835449

[115] Buckner RL Andrews-Hanna JR Schacter DL The brain’s default network: anatomy, function, and relevance to disease. Ann N Y Acad Sci (2008) 1124:1–38. 10.1196/annals.1440.011 18400922

[116] Corbetta M Patel G Shulman GL The reorienting system of the human brain: from environment to theory of mind. Neuron (2008) 58:306–24. 10.1016/j.neuron.2008.04.017 PMC2441869 18466742

[117] Uddin LQ Salience processing and insular cortical function and dysfunction. Nat Rev Neurosci (2015) 16:55–61. 10.1038/nrn3857 25406711

[118] Nair A Jolliffe M Lograsso YSS Bearden CE. A review of default mode network connectivity and its association with social cognition in adolescents with autism spectrum disorder and early-onset psychosis. Front Psychiatry (2020) 11:614. 32670121 10.3389/fpsyt.2020.00614 PMC7330632

[119] Dosenbach NUF Visscher KM Palmer ED Miezin FM Wenger KK Kang HC A core system for the implementation of task sets. Neuron (2006) 50:799–812. 10.1016/j.neuron.2006.04.031 16731517 PMC3621133

[120] Barrett LF Satpute AB Large-scale brain networks in affective and social neuroscience: towards an integrative functional architecture of the brain. Curr Opin Neurobiol (2013) 23:361–72. 10.1016/j.conb.2012.12.012 PMC4119963 23352202

[121] Bartholomeusz CF Ganella EP Labuschagne I Bousman C Pantelis C Effects of oxytocin and genetic variants on brain and behaviour: Implications for treatment in schizophrenia. Schizophr Res (2015) 168:614–27. 10.1016/j.schres.2015.06.007 26123171

[122] Andari E Hurlemann R Young LJ A Precision Medicine Approach to Oxytocin Trials. Curr Top Behav Neurosci (2018) 35:559–90. 10.1007/7854_2017_29 PMC5815942 28812273

[123] Luo S Ma Y Liu Y Li B Wang C Shi Z Interaction between oxytocin receptor polymorphism and interdependent culture values on human empathy. Soc Cognit Affect Neurosci (2015) 10:1273–81. 10.1093/scan/nsv019 PMC4560951 25680993

[124] Rodrigues SM Saslow LR Garcia N John OP Keltner D Oxytocin receptor genetic variation relates to empathy and stress reactivity in humans. Proc Natl Acad Sci U.S.A. (2009) 106:21437–41. 10.1073/pnas.0909579106 PMC2795557 19934046

[125] Smith KE Porges EC Norman GJ Connelly JJ Decety J Oxytocin receptor gene variation predicts empathic concern and autonomic arousal while perceiving harm to others. Soc Neurosci (2014) 9:1–9. 10.1080/17470919.2013.863223 24295535 PMC3923324

[126] Uzefovsky F Shalev I Israel S Edelman S Raz Y Mankuta D Oxytocin receptor and vasopressin receptor 1a genes are respectively associated with emotional and cognitive empathy. Horm Behav (2015) 67:60–5. 10.1016/j.yhbeh.2014.11.007 25476609

[127] Wu N Li Z Su Y The association between oxytocin receptor gene polymorphism (OXTR) and trait empathy. J Affect Disord (2012) 138:468–72. 10.1016/j.jad.2012.01.009 22357335

[128] Krueger F Parasuraman R Iyengar V Thornburg M Weel J Lin M Oxytocin receptor genetic variation promotes human trust behavior. Front Hum Neurosci (2012) 6:4. 10.3389/fnhum.2012.00004 22347177 PMC3270329

[129] Tost H Kolachana B Hakimi S Lemaitre H Verchinski BA Mattay VS A common allele in the oxytocin receptor gene (OXTR) impacts prosocial temperament and human hypothalamic-limbic structure and function. Proc Natl Acad Sci U.S.A. (2010) 107:13936–41. 10.1073/pnas.1003296107 PMC2922278 20647384

[130] Baribeau DA Dupuis A Paton TA Scherer SW Schachar RJ Arnold PD Oxytocin Receptor Polymorphisms are Differentially Associated with Social Abilities across Neurodevelopmental Disorders. Sci Rep (2017) 7:11618. 10.1038/s41598-017-10821-0 28912494 PMC5599599

[131] Harrison AJ Gamsiz ED Berkowitz IC Nagpal S Jerskey BA Genetic variation in the oxytocin receptor gene is associated with a social phenotype in autism spectrum disorders. Am J Med Genet Part B Neuropsychiatr Genet (2015) 168:720–9. 10.1002/ajmg.b.32377 26365303

[132] Wade M Hoffmann TJ Wigg K Jenkins JM Association between the oxytocin receptor (OXTR) gene and children’s social cognition at 18 months. Genes Brain Behav (2014) 13:603–10. 10.1111/gbb.12148 24916666

[133] Westberg L Henningsson S Zettergren A Svärd J Hovey D Lin T Variation in the Oxytocin Receptor Gene Is Associated with Face Recognition and its Neural Correlates. Front Behav Neurosci (2016) 10:178. 10.3389/fnbeh.2016.00178 27713694 PMC5031602

[134] Wilczyński KM Siwiec A Janas-Kozik M Systematic Review of Literature on Single-Nucleotide Polymorphisms Within the Oxytocin and Vasopressin Receptor Genes in the Development of Social Cognition Dysfunctions in Individuals Suffering From Autism Spectrum Disorder. Front Psychiatry (2019) 10:380. 10.3389/fpsyt.2019.00380 31214061 PMC6554290

[135] Zhao W Luo R Sindermann C Li J Wei Z Zhang Y Oxytocin modulation of self-referential processing is partly replicable and sensitive to oxytocin receptor genotype. Prog Neuropsychopharmacol Biol Psychiatry (2020) 96:109734. 10.1016/j.pnpbp.2019.109734 31415827

[136] Quintana DS Guastella AJ An Allostatic Theory of Oxytocin. Trends Cognit Sci (2020) 24:515–28. 10.1016/j.tics.2020.03.008 32360118

[137] Jack A Connelly JJ Morris JP DNA methylation of the oxytocin receptor gene predicts neural response to ambiguous social stimuli. Front Hum Neurosci (2012) 6:280. 10.3389/fnhum.2012.00280 23087634 PMC3467966

[138] Puglia MH Connelly JJ Morris JP Epigenetic regulation of the oxytocin receptor is associated with neural response during selective social attention. Transl Psychiatry (2018) 8:116. 10.1038/s41398-018-0159-x 29907738 PMC6003910

[139] Andari E Nishitani S Kaundinya G Caceres GA Morrier MJ Ousley O Epigenetic modification of the oxytocin receptor gene: implications for autism symptom severity and brain functional connectivity. Neuropsychopharmacol (2020) 45(7):1150–8. 10.1038/s41386-020-0610-6 PMC7235273 31931508

[140] Gregory SG Connelly JJ Towers AJ Johnson J Biscocho D Markunas CA Genomic and epigenetic evidence for oxytocin receptor deficiency in autism. BMC Med (2009) 7:62. 10.1186/1741-7015-7-62 19845972 PMC2774338

[141] Aydın O Lysaker PH Balıkçı K Ünal-Aydın P Esen-Danacı A Associations of oxytocin and vasopressin plasma levels with neurocognitive, social cognitive and meta cognitive function in schizophrenia. Psychiatry Res (2018) 270:1010–6. 10.1016/j.psychres.2018.03.048 29609987

[142] Strauss GP Chapman HC Keller WR Koenig JI Gold JM Carpenter WT Endogenous oxytocin levels are associated with impaired social cognition and neurocognition in schizophrenia. J Psychiatr Res (2019) 112:38–43. 10.1016/j.jpsychires.2019.02.017 30849617

[143] Aspé-Sánchez M Moreno M Rivera MI Rossi A Ewer J Oxytocin and Vasopressin Receptor Gene Polymorphisms: Role in Social and Psychiatric Traits. Front Neurosci (2015) 9:510. 10.3389/fnins.2015.00510 26858594 PMC4729929

[144] Carson DS Garner JP Hyde SA Libove RA Berquist SW Hornbeak KB Arginine Vasopressin Is a Blood-Based Biomarker of Social Functioning in Children with Autism. PloS One (2015) 10:e0132224. 10.1371/journal.pone.0132224 26200852 PMC4511760

[145] Walter NT Markett SA Montag C Reuter M A genetic contribution to cooperation: dopamine-relevant genes are associated with social facilitation. Soc Neurosci (2011) 6:289–301. 10.1080/17470919.2010.527169 21061227

[146] Reuter M Frenzel C Walter NT Markett S Montag C Investigating the genetic basis of altruism: the role of the COMT Val158Met polymorphism. Soc Cognit Affect Neurosci (2011) 6:662–8. 10.1093/scan/nsq083 PMC3190209 21030481

[147] Herrmann MJ Würflein H Schreppel T Koehler S Mühlberger A Reif A Catechol-O-methyltransferase Val158Met genotype affects neural correlates of aversive stimuli processing. Cognit Affect Behav Neurosci (2009) 9:168–72. 10.3758/CABN.9.2.168 19403893

[148] Swart M Bruggeman R Larøi F Alizadeh BZ Kema I Kortekaas R COMT Val158Met polymorphism, verbalizing of emotion and activation of affective brain systems. NeuroImage (2011) 55:338–44. 10.1016/j.neuroimage.2010.12.017 21156209

[149] Williams LM Gatt JM Grieve SM Dobson-Stone C Paul RH Gordon E COMT Val(108/158)Met polymorphism effects on emotional brain function and negativity bias. NeuroImage (2010) 53:918–25. 10.1016/j.neuroimage.2010.01.084 20139013

[150] Poletti S Radaelli D Cavallaro R Bosia M Lorenzi C Pirovano A Catechol-O-methyltransferase (COMT) genotype biases neural correlates of empathy and perceived personal distress in schizophrenia. Compr Psychiatry (2013) 54:181–6. 10.1016/j.comppsych.2012.06.008 22901597

[151] Tylec A Jeleniewicz W Mortimer A Bednarska-Makaruk M Kucharska K Interaction Between Val158Met Catechol-O-Methyltransferase Polymorphism and Social Cognitive Functioning in Schizophrenia: Pilot Study. Ann Hum Genet (2017) 81:267–75. 10.1111/ahg.12209 28856668

[152] Dunne PW Roberts DL Quinones MP Velligan DI Paredes M Walss-Bass C Immune markers of social cognitive bias in schizophrenia. Psychiatry Res (2017) 251:319–24. 10.1016/j.psychres.2017.02.030 28237910

[153] Moieni M Eisenberger NI Effects of inflammation on social processes and implications for health. Ann N Y Acad Sci (2018) 1428:5–13. 10.1111/nyas.13864 29806109 PMC6158086

[154] Rose EJ Hargreaves A Morris D Fahey C Tropea D Cummings E Effects of a novel schizophrenia risk variant rs7914558 at CNNM2 on brain structure and attributional style. Br J Psychiatry J Ment Sci (2014) 204:115–21. 10.1192/bjp.bp.113.131359 24311551

[155] Tsang SY Zhong S Mei L Chen J Ng S-K Pun FW Social cognitive role of schizophrenia candidate gene GABRB2. PloS One (2013) 8:e62322. 10.1371/journal.pone.0062322 23638040 PMC3634734

[156] Frascarelli M Padovani G Buzzanca A Accinni T Carlone L Ghezzi F Social cognition deficit and genetic vulnerability to schizophrenia in 22q11 deletion syndrome. Ann Ist Super Sanita (2020) 56:107–13. 10.4415/ANN_20_01_15 32242542

[157] Jalbrzikowski M Carter C Senturk D Chow C Hopkins JM Green MF Social cognition in 22q11.2 microdeletion syndrome: relevance to psychosis? Schizophr Res (2012) 142:99–107. 10.1016/j.schres.2012.10.007 23122739 PMC3714207

[158] Lattanzi GM Buzzanca A Frascarelli M Di Fabio F Genetic and clinical features of social cognition in 22q11.2 deletion syndrome. J Neurosci Res (2018) 96:1631–40. 10.1002/jnr.24265 30004142

[159] Lin A Vajdi A Kushan-Wells L Helleman G Hansen LP Jonas RK Reciprocal Copy Number Variations at 22q11.2 Produce Distinct and Convergent Neurobehavioral Impairments Relevant for Schizophrenia and Autism Spectrum Disorder. Biol Psychiatry (2020) 88(3):260–72. 10.1016/j.biopsych.2019.12.028 PMC7354903 32143830

[160] Norkett EM Lincoln SH Gonzalez-Heydrich J D’Angelo EJ Social cognitive impairment in 22q11 deletion syndrome: A review. Psychiatry Res (2017) 253:99–106. 10.1016/j.psychres.2017.01.103 28364592

[161] Shashi V Harrell W Eack S Sanders C McConkie-Rosell A Keshavan MS Social cognitive training in adolescents with chromosome 22q11.2 deletion syndrome: feasibility and preliminary effects of the intervention. J Intellect Disabil Res JIDR (2015) 59:902–13. 10.1111/jir.12192 PMC5824427 25871427

[162] Erk S Mohnke S Ripke S Lett TA Veer IM Wackerhagen C Functional neuroimaging effects of recently discovered genetic risk loci for schizophrenia and polygenic risk profile in five RDoC subdomains. Transl Psychiatry (2017) 7:e997. 10.1038/tp.2016.272 28072415 PMC5545733

[163] Hayes GS McLennan SN Henry JD Phillips LH Terrett G Rendell PG Task characteristics influence facial emotion recognition age-effects: A meta-analytic review. Psychol Aging (2020) 35:295–315. 10.1037/pag0000441 31999152

[164] Kordsachia CC Labuschagne I Stout JC Beyond emotion recognition deficits: A theory guided analysis of emotion processing in Huntington’s disease. Neurosci Biobehav Rev (2017) 73:276–92. 10.1016/j.neubiorev.2016.11.020 27913281

[165] Grace SA Rossell SL Heinrichs M Kordsachia C Labuschagne I Oxytocin and brain activity in humans: A systematic review and coordinate-based meta-analysis of functional MRI studies. Psychoneuroendocrinology (2018) 96:6–24. 10.1016/j.psyneuen.2018.05.031 29879563

[166] Erdozain AM Peñagarikano O Oxytocin as Treatment for Social Cognition, Not There Yet. Front Psychiatry (2020) 10:930. 10.3389/fpsyt.2019.00930 31998152 PMC6962227

